# Measures of repolarization variability predict ventricular arrhythmogenesis in heptanol-treated Langendorff-perfused mouse hearts

**DOI:** 10.1016/j.crphys.2021.04.001

**Published:** 2021-04-19

**Authors:** Gary Tse, Guoliang Hao, Sharen Lee, Jiandong Zhou, Qingpeng Zhang, Yimei Du, Tong Liu, Shuk Han Cheng, Wing Tak Wong

**Affiliations:** a Tianjin Key Laboratory of Ionic-Molecular Function of Cardiovascular Disease, Department of Cardiology, Tianjin Institute of Cardiology, Second Hospital of Tianjin Medical University, Tianjin, 300211, China; b Cardiovascular Analytics Group, Laboratory of Cardiovascular Physiology, Hong Kong, China; c Department of Physiology, Anatomy and Genetics, University of Oxford, Oxford, United Kingdom; d School of Data Science, City University of Hong Kong, Hong Kong, China; e Research Center of Ion Channelopathy, Institute of Cardiology, Union Hospital, Tongji Medical College, Huazhong University of Science and Technology, Wuhan, China; f Department of Biomedical Sciences, City University of Hong Kong, Tat Chee Avenue, Kowloon Tong, Hong Kong; g School of Life Sciences, Chinese University of Hong Kong, Hong Kong, China

**Keywords:** Action potential duration, Variability, Entropy, Detrended fluctuation analysis, Heptanol

## Abstract

**Background:**

Time-domain and non-linear methods can be used to quantify beat-to-beat repolarization variability but whether measures of repolarization variability can predict ventricular arrhythmogenesis in mice have never been explored.

**Methods:**

Left ventricular monophasic action potentials (MAPs) were recorded during constant right ventricular 8 ​Hz pacing in Langendorff-perfused mouse hearts, in the presence or absence of the gap junction and sodium channel inhibitor heptanol (0.1, 0.5, 1 or 2 ​mM).

**Results:**

Under control conditions, mean action potential duration (APD) was 39.4 ​± ​8.1 ​ms. Standard deviation (SD) of APDs was 0.3 ​± ​0.2 ​ms, coefficient of variation was 0.9 ​± ​0.8% and the root mean square (RMS) of successive differences in APDs was 0.15 ​± ​0.14 ​ms. Poincaré plots of APD_n+1_ against APD_n_ revealed ellipsoid morphologies with a SD along the line-of-identity (SD2) to SD perpendicular to the line-of-identity (SD1) ratio of 4.6 ​± ​2.1. Approximate and sample entropy were 0.61 ​± ​0.12 and 0.76 ​± ​0.26, respectively. Detrended fluctuation analysis revealed short- and long-term fluctuation slopes of 1.49 ​± ​0.27 and 0.81 ​± ​0.36, respectively. Heptanol at 2 ​mM induced ventricular tachycardia in five out of six hearts. None of the above parameters were altered by heptanol during which reproducible electrical activity was observed (KW-ANOVA, *P* ​> ​0.05). Contrastingly, SD2/SD1 decreased to 2.03 ​± ​0.41, approximate and sample entropy increased to 0.82 ​± ​0.12 and 1.45 ​± ​0.34, and short-term fluctuation slope decreased to 0.82 ​± ​0.19 during the 20-s period preceding spontaneous ventricular tachy-arrhythmias (n ​= ​6, KW-ANOVA, P ​< ​0.05).

**Conclusion:**

Measures of repolarization variability, such as SD2/SD1, entropy, and fluctuation slope are altered preceding the occurrence of ventricular arrhythmogenesis in mouse hearts. Changes in these variables may allow detection of impending arrhythmias for early intervention.

## Highlights

• None of the parameters on variability determined using time-domain or non-linear analyses was altered by heptanol.
• In the immediate periods preceding the onset of spontaneous ventricular tachycardia, the following changes were observed.
• SD2/SD1 was decreased, approximate and sample entropy were increased, and short-term fluctuation slope was decreased.
• These measures of repolarization variability could be used to predict impending ventricular arrhythmias in mouse hearts.

## Graphical abstract

**Figure:**
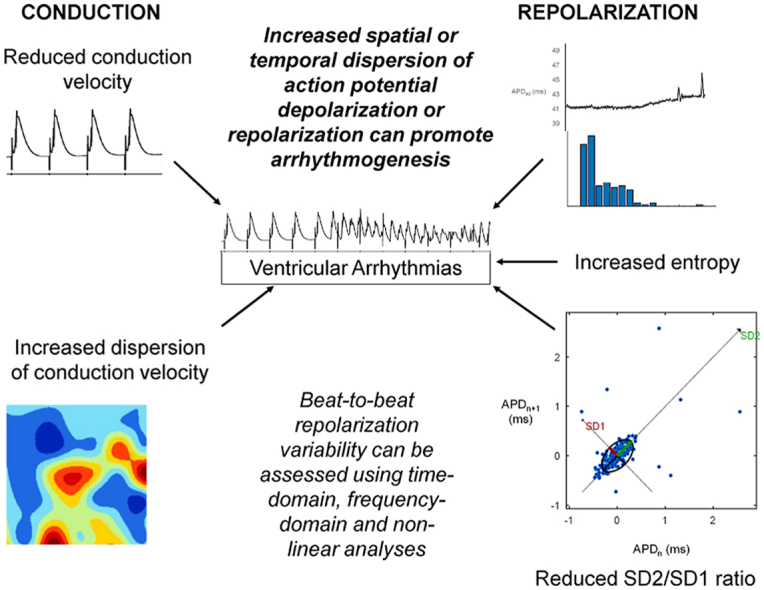

## 1 Introduction

Beat-to-beat variations in the repolarization time-course is an inherent property of cardiac electrophysiological function (Couderc, 2009). This may be observed as variability of action potential durations (APDs) at the single cell level (Nanasi et al., 2017), or of QT durations at the systems level (Niemeijer et al., 2014; Phadumdeo and Weinberg, 2018; Orini et al., 2016; Nollo et al., 1992). APD variability can be affected by different physiological states, such as the extent of intercellular coupling (Zaniboni et al., 2000), redox changes (Kistamas et al., 2015a), abnormal calcium dynamics (Kistamas et al., 2015b) or time taken for full repolarization (Abi-Gerges et al., 2010). Recently, our team reported the use of both time-domain and non-linear analyses to quantify APD variability for the first time in mouse hearts (Tse et al., 2018). Theoretically, the loss of intercellular coupling can increase this variability (Magyar et al., 2015), which could precipitate arrhythmogenesis. Heptanol, a pharmacological agent that inhibits gap junctions and sodium channels at 2 ​mM or above, is known to exert ventricular pro-arrhythmic effects under different experimental conditions (Callans et al., 1996; Tse et al., 2012, 2016a). In our previous work, we attributed its arrhythmogenic effects in mice to conduction abnormalities alone. However, conduction parameters could not distinguish arrhythmic from refractory outcomes. For example, conduction velocity was reduced by similar extents in arrhythmic and non-arrhythmic hearts, which would suggest other factors were predisposing to arrhythmogenesis. Since our previous analysis simply used the mean duration of repolarization without assessing its beat-to-beat variability, in this study we tested the hypothesis that repolarization variability by non-linear measures can predict heptanol-induced ventricular arrhythmias in Langendorff-perfused mouse hearts.

## 2 Materials and methods

### 2.1 Solutions

Krebs-Henseleit solution (composition in mM: NaCl 119, NaHCO_3_ 25, KCl 4, KH_2_PO_4_ 1.2, MgCl_2_ 1, CaCl_2_ 1.8, glucose 10 and sodium pyruvate 2, pH 7.4), which has been bicarbonate-buffered and bubbled with 95% O_2_–5% CO_2_, was used in the experiments described in this study. Heptanol (Sigma, Dorset, UK; density: 0.82 ​g ​ml^−1^) is an agent that remains soluble in aqueous solutions up to 9 ​mM (The Merck Index, New Jersey, USA). Krebs-Henseleit solution was used to dilute the heptanol solution to produce a final concentration of 0.05 and 2 ​mM.

### 2.2 Preparation of Langendorff-perfused mouse hearts

This study was approved by the Animal Welfare and Ethical Review Body at the University of Cambridge. Wild-type mice of 129 genetic background between 5 and 7 months of age were used. They were maintained at room temperature (21 ​± ​1 ​°C) and were subjected to a 12:12 ​h light/dark cycle with free access to sterile rodent chow and water in an animal facility. Mice were terminated by dislocation of the cervical spine in accordance with Sections 1, 2 of Schedule 1 of the UK Animals (Scientific Procedures) Act 1986. After removal from their chest cavities, the hearts were submerged in ice-cold Krebs-Henseleit solution. The aortas were cannulated using a custom-made 21-gauge cannula prefilled with ice-cold buffer. A micro-aneurysm clip (Harvard Apparatus, UK) was used to secure the hearts onto the Langendorff perfusion system. Retrograde perfusion was carried out at a flow rate of 2–2.5 ​ml ​min^−1^ by use of a peristaltic pump (Watson–Marlow Bredel pumps model 505S, Falmouth, Cornwall, UK). The perfusate passed through successively 200 and 5 ​μm filters and warmed to 37 ​°C using a water jacket and circulator before arriving at the aorta. Approximately 90% of the hearts regained their pink colour and spontaneous rhythmic activity. These were therefore studied further. The remaining 10% did not and were discarded. The hearts were perfused for a further 20 ​min to minimise residual effects of endogenous catecholamine release, before their electrophysiology properties were characterized.

### 2.3 Stimulating and recording procedures

Paired platinum electrodes (1 ​mm interpole distance) were used to stimulate the right ventricular epicardium electrically. This took place at 8 ​Hz, using square wave pulses of 2 ​ms in duration, with a stimulation voltage set to three times the diastolic threshold (Grass S48 Stimulator, Grass-Telefactor, Slough, UK) immediately after the start of perfusion.

A monophasic action potential (MAP) electrode was used to record MAPs from the left ventricular epicardium (Linton Instruments, Harvard Apparatus). The stimulating and recording electrodes were maintained at constant positions separated approximately by distance of 3 ​mm. All recordings were performed using a baseline cycle length (BCL) of 125 ​ms (8 ​Hz) to exclude rate-dependent differences in action potential durations (APDs). This frequency was selected because it is within the range of the normal *in vivo* heart rate. MAPs were pre-amplified using a NL100AK head stage, amplified with a NL 104A amplifier and band pass filtered between 0.5 ​Hz and 1 ​kHz using a NL125/6 filter (Neurolog, Hertfordshire, UK) and then digitized (1401plus MKII, Cambridge Electronic Design, Cambridge, UK) at 5 ​kHz. Waveforms were analysed using Spike2 software (Cambridge Electronic Design, UK). MAP waveforms that did not match established criteria for MAP signals were rejected (Knollmann et al., 2001; Tse et al., 2016b). They must have reproducible baselines, fast upstrokes, with no inflections or negative spikes, and a rapid first phase of repolarization. 0% repolarization was measured at the peak of the MAP and 100% repolarization was measured at the point of return of the potential to baseline (Knollmann et al., 2001; Gussak et al., 2000; Fabritz et al., 2003).

### 2.4 APD variability analysis

APD variability analysis was performed using Kubios HRV Standard software (Version 3.0.2) over a 20-s period. Time-domain analysis yielded the 1) standard deviation (SD) of APDs, which represents the overall (short-term and long-term) variability, and 2) root mean square (RMSSD) of successive differences of APDs, which represents the short-term variability:

$$\text{SDAPD ​}=\sqrt{\frac{1}{N-1}\overset{N}{\underset{j=1}{\sum }}{\left(APD_{j}-\;A\bar{PD}\right)}^{2}}$$

$$\text{RMSSD ​}=\sqrt{\frac{1}{N-1}\overset{N-1}{\underset{j=1}{\sum }}{\left(APD_{j+1}-APD_{j}\right)}^{2}}$$

Frequency-domain analysis was conducted using the Fast Fourier Transform method. For frequency domain parameters, spectral analysis was performed by using fast-Fourier transform method. The sampling frequency was set to 8 ​Hz. The power in the repolarization spectrum between 0.04 and 4 ​Hz was defined as total power (TP). The power in the repolarization spectrum was divided into three different frequency bands: very low frequency power (VLF, 0–0.04 ​Hz), low frequency power (LF, 0.04–1.5 ​Hz) and high frequency power (HF, 1.5–4 ​Hz).

The above frequency analysis does not provide any information on the time evolution of the frequencies. To achieve, this, time-frequency analysis was conducted using two different techniques. Firstly, short-time Fourier transform (STFT) was used to break the signal into small time segments using an appropriate sliding-window function, and then apply a Fourier transformation to the successive sliding-window segments. The Hanning window with a Fast Fourier Transform length of 256 and overlap of 128 were selected.

Secondly, continuous wavelet transform (CWT) was used to divide a continuous-time function into wavelets given by:

$$\text{CWT}\left(\text{a,b}\right)\text{ ​}=\frac{1}{\sqrt{a}}\;\overset{+\infty }{\underset{-\;\infty }{\int }}x\left(t\right)\;.\;\psi^{*}\left(\frac{t-b}{a}\right)dt$$

Where the superscript, ∗, is the complex conjugate and ψ_a,b_∗ represents a translated and scaled complex conjugated mother wavelet. The mother wavelet ψ is invertible when it verifies the condition of admissibility which is stated as:

$$\overset{+\infty }{\underset{-\infty }{\int }}\frac{\left|\hat{\psi }\left(\omega \right)\right|}{\omega }\;d\omega <\infty$$

The Morlet wavelet was selected, which uses a Gaussian-modulated sinusoid:

$$\psi \left(t\right)=\;\frac{1}{\sqrt[{4}]{\pi }}\left(e^{i\omega_{o}t}-\;e^{-\frac{\omega_{o}^{2}}{2}}\right)\;e^{-\frac{t^{2}}{2}}$$

where ω_o_ is the central frequency of the mother wavelet. The second term in the brackets corrects for the non-zero mean of the complex sinusoid of the first term. This becomes negligible for values of ω_o_ ​> ​5, which we selected in our case:

$$\psi \left(t\right)=\;\frac{1}{\sqrt[{4}]{\pi }}e^{i\omega_{o}t}\;e^{-\frac{t^{2}}{2}}$$

Non-linear properties of APD variability were studied as follow. Poincaré plots are graphical representations of the correlation between successive APD values, in which APD_n+1_ is plotted against APD_n_. This enables determination of the SD of the points perpendicular to the line-of-identity (SD1). Different points along this perpendicular axis represent a beat-to-beat variation between the initial (n) and subsequent (n+1) contraction, representing multiple two-beat ‘snapshots’ with little correlation to a progressive time parameter. Therefore, SD1 is associated with instantaneous or short-term variability. As for the points along the line-of-identity (SD2), it shows beat-to-beat consistency between the initial (n) and subsequent (n+1) RR interval. Hence, deviation of the clustered SD2 points away from the average RR interval, taken with reference to the centroid, represents long-term variability. The ratio SD2 to SD1 then gives an indication of the degree of long-term variability in relation to the short-term variability.

Coined in 1991 by Pincus et al., the concept of approximate entropy was introduced to provide approximations on the degree of regularity when applied to a short-duration epoch, which cannot be achieved with moment statistics such as mean and variance. This is applied to non-stationary biomedical data such as heart rate variability, which commonly presents with non-linearity and complexity. Logarithmically, the approximate entropy takes into account the imputed threshold ‘*r’* under which a recurrence is identified. With this it expresses the likelihood of repeated signals within the threshold for *m* and *m*+1 points. It is computed as follows:

Firstly, a set of length m vectors u_j_ is formed:

u_j_ = (APD_j_; APD_j+1_, …, APD_j+m-1_); j ​= ​1; 2; … N – m ​+ ​1

where m is the embedding dimension and N is the number of measured APDs. The distance between these vectors is defined as the maximum absolute difference between the corresponding elements:

d(u_j_, u_k_) ​= ​max {|APD_j+n_ – APD_k+n_| |n ​= ​0, …, m-1}

for each u_j_ the relative number of vectors u_k_ for which d(u_j_, u_k_) ​≤ ​r is calculated. This index is denoted with Cmj (r) and can be written in the form

$$C_{j}^{m}\left(r\right)=\;\frac{nbr\;of\;\left\{u_{k}|\;d\left(u_{j},u_{k}\right)\leq r\right\}}{N-m+1}\;\forall k$$

Taking the natural logarithms gives:

$${\text{Φ}}^{m}\left(r\right)=\;\frac{1}{N-m+1}\;\overset{N-m+1}{\underset{j=1}{\sum }}\ln\;C_{j}^{m}\left(r\right).$$

The approximate entropy is then defined as:

$$ApEn\;\left(m,\;r,\;N\right)=\;{\text{Φ}}^{m}\left(r\right)-\;{\text{Φ}}^{m+1}\left(r\right)$$

Approximate entropy measures the likelihood that certain patterns of observations are followed by different patterns of observations. As such, a lower approximate entropy values reflect a more regular signal, whereas higher values reflect a more irregular signal (Mesin, 2018; Pincus, 1991).

The sample entropy also provides a measure of signal irregularity but is less susceptible to bias than approximate entropy (Richman and Moorman, 2000; Nayak et al., 2018). This is done by eliminating the counting of self-matches; hence the count of the number of similar vector lengths is always one less than that of ApEn. Furthermore, sample entropy uses the logarithm of the sum of conditional properties rather than each conditional property individually, illustrated by the negative natural logarithm for conditional properties. Both sample entropy and approximate entropy are able to differentiate between experimental and theoretical data sets. However, it has been demonstrated that sample entropy yielded better relative consistency compared to approximate entropy, reflecting independence from data length and choice of *m* or *r* (Molina-Pico et al., 2011).

This is given by:

$$C_{j}^{m}\left(r\right)=\;\frac{nbr\;of\;\left\{u_{k}|\;d\left(u_{j},u_{k}\right)\leq r\right\}}{N-m}\;\forall k\;\neq j$$

Averaging then gives:

$$C^{m}\left(r\right)=\;\frac{1}{N-m+1}\;\overset{N-m+1}{\underset{j=1}{\sum }}C_{j}^{m}\left(r\right)$$

The sample entropy is then given by:

$$\text{SampEn}\left(m,\;r,\;N\right)=\;\ln(\frac{C^{m}\left(r\right)}{C^{m+1}\left(r\right)})$$

Finally, detrended fluctuation analysis (DFA) was performed to determine long-range correlations in non-stationary physiological time series (Peng et al., 1995), yielding both short-term fluctuation (α1) and long-term fluctuation (α2) slopes. The point at which the slopes α1 and α2 is the crossover point.

### 2.5 Statistical analysis

All values were expressed as mean ​± ​standard error of the mean (SEM). Numerical data were compared by Kruskal-Wallis analysis of variance (KW-ANOVA). *P* ​< ​0.05 was considered statistically significant and was denoted by ∗ in the figures.

## 3 Results

### 3.1 Action potential duration variability determined using time-domain methods

**Fig. 1:**
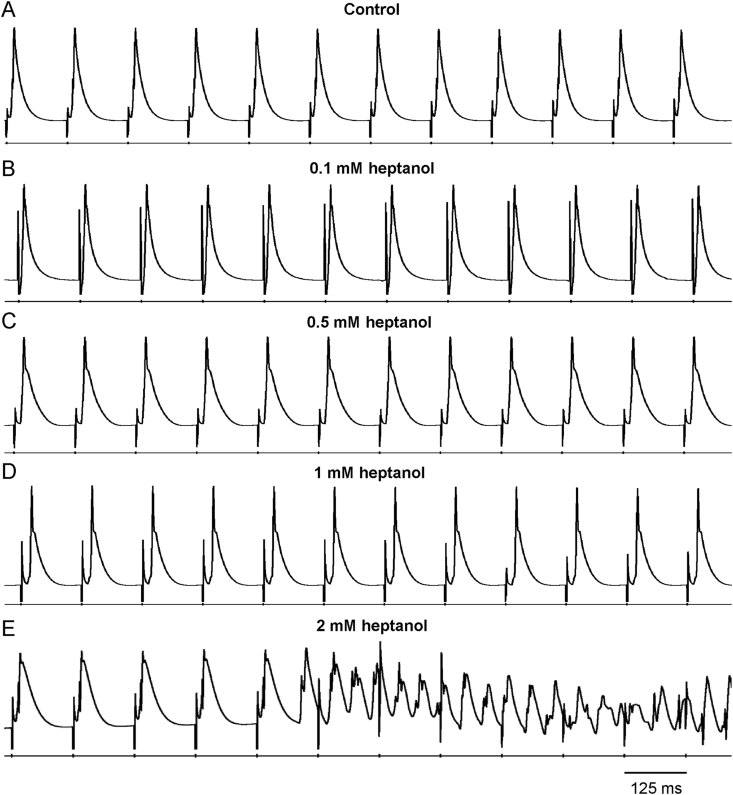
MAP traces from a representative heart during regular 8 ​Hz pacing before (A) or after the application of 0.1 (B), 0.5 (C), 1 (D) or 2 (E) mM heptanol.

**Fig. 2:**
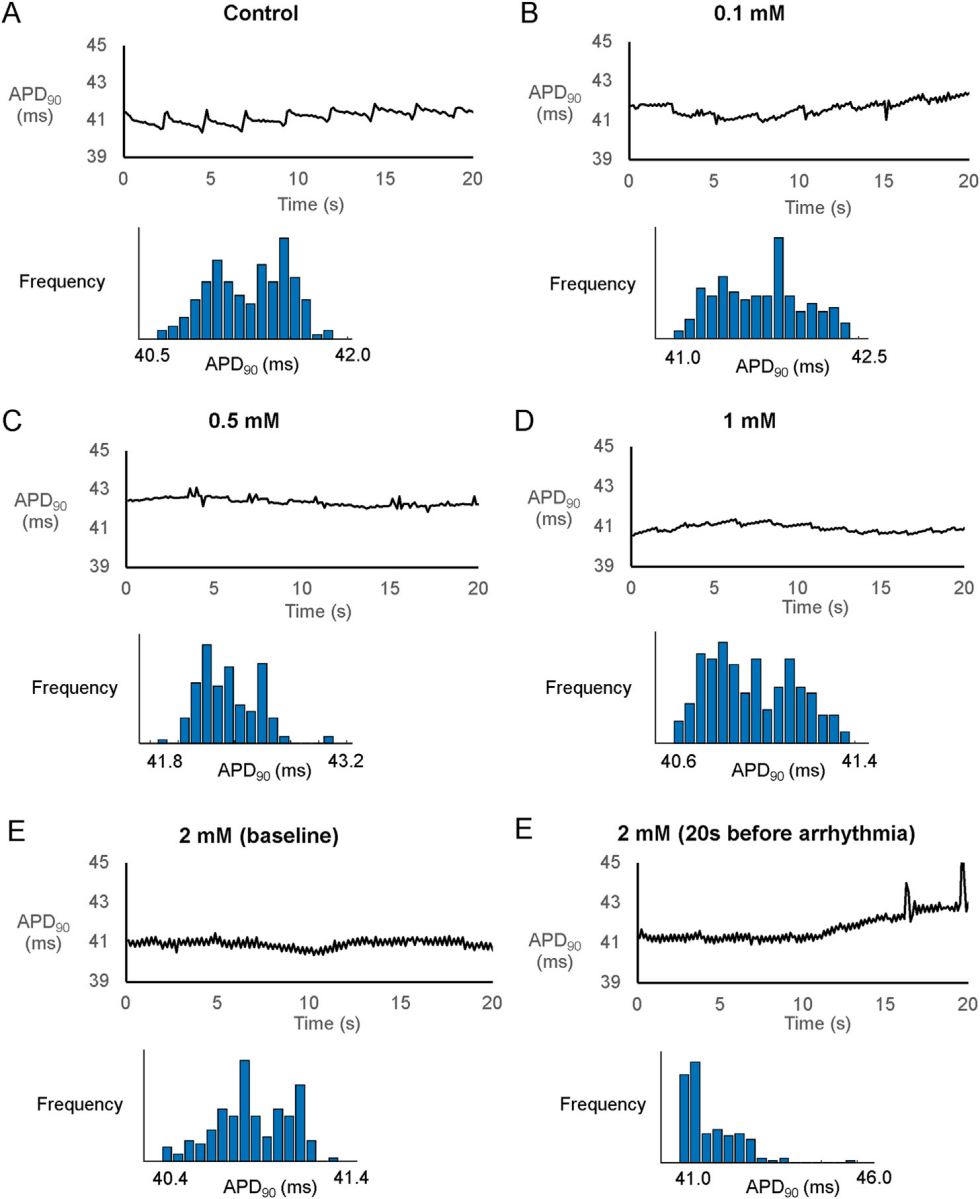
Time series and histograms of APDs from a representative heart before (A) or after the application of 0.1 (B), 0.5 (C), 1 (D) or 2 (E) mM heptanol.

**Fig. 3:**
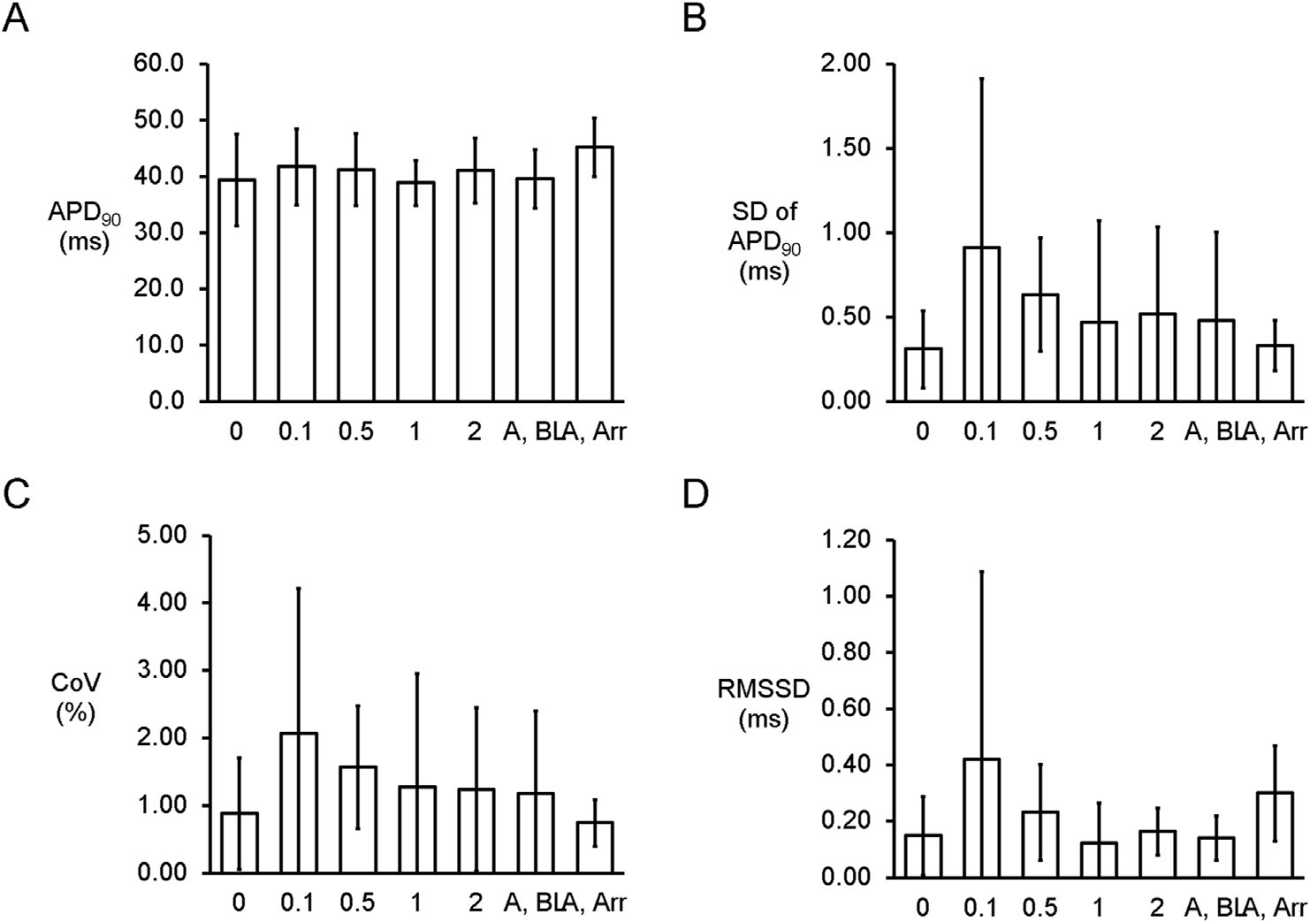
Time-domain analysis yielding mean APD (A), standard deviation (SD) of APDs (B), coefficient of variation (CoV) (C), and root mean square (RMS) of successive differences of APDs (D) (n ​= ​6) in the presence or absence of heptanol.

Reproducible MAP recordings were obtained from the left ventricular epicardium during regular pacing of the right ventricular epicardium at 8 ​Hz in Langendorff-perfused mouse hearts (n ​= ​6 hearts). The representative tracings before and after the application of heptanol at 0.1, 0.5, 1 and 2 ​mM are shown in Fig. 1A–E. Heptanol at 2 ​mM, but not at lower concentrations, significantly increased the incidence of ventricular arrhythmias occurring spontaneously compared to baseline (0/6 hearts vs. 5/6 hearts respectively; *P* ​< ​0.05, Fisher’s exact test). The typical time series and histograms of action potential durations (APDs) at 90% repolarization are shown in Fig. 2A–E, respectively. Time-domain analysis demonstrated a mean APD of 39.4 ​± ​8.1 ​ms (Fig. 3A), standard deviation (SD) of APDs of 0.31 ​± ​0.23 ​ms (Fig. 3B), coefficient of variation (CoV) of 0.88 ​± ​0.82% (Fig. 3C), and root mean square (RMS) of successive differences in APDs of 0.15 ​± ​0.14 ​ms (Fig. 3D). None of these parameters were altered by heptanol at the concentrations studied during the periods where reproducible MAPs were recorded, nor during the 20 ​s that preceded the onset of ventricular arrhythmias (KW-ANOVA, *P* ​> ​0.05).

### 3.2 Action potential duration variability determined using non-linear methods

**Fig. 4:**
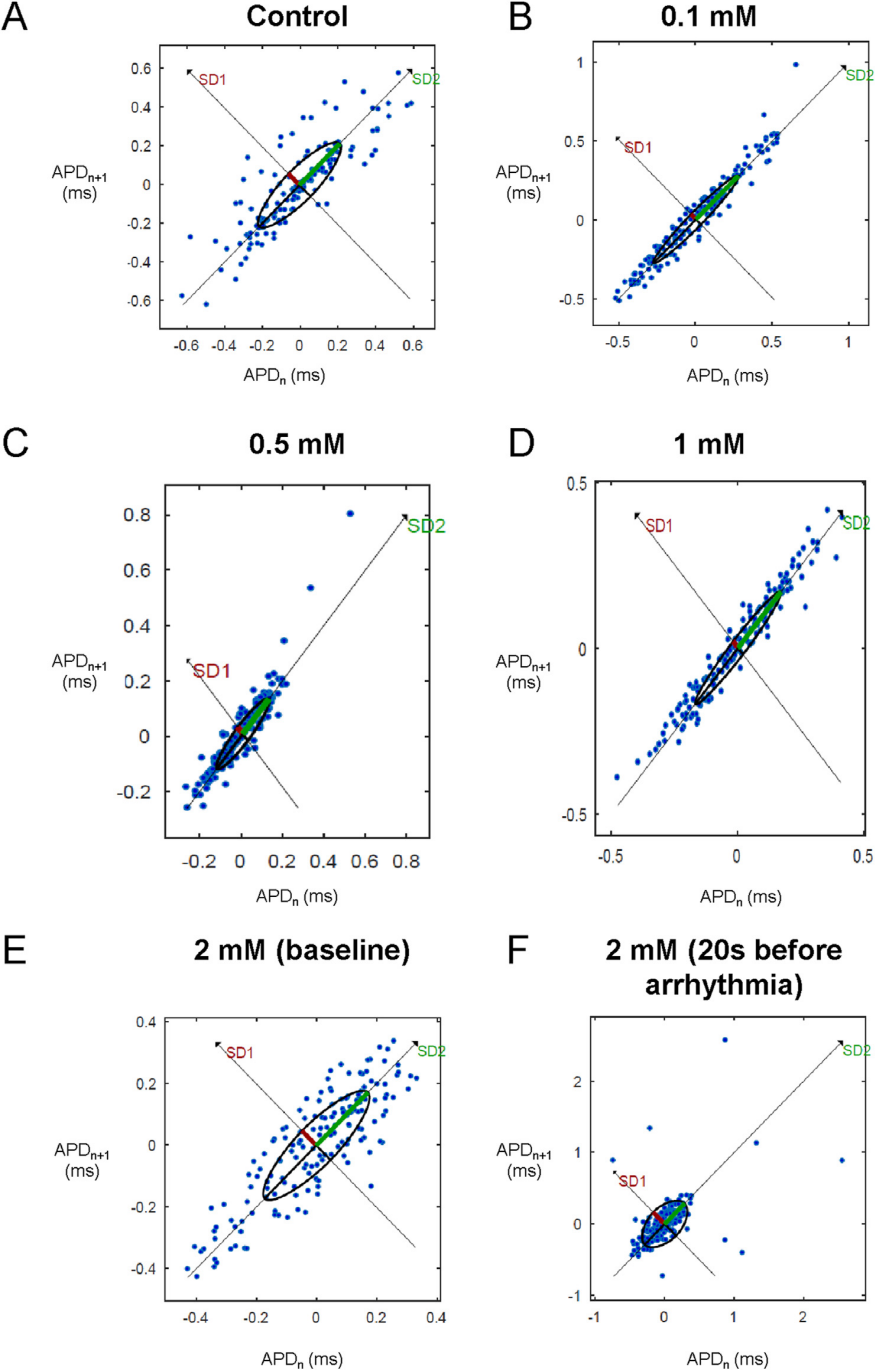
Representative Poincaré plots of APD_n+1_ against APD_n_ with SD along the line-of-identity (SD1) and SD perpendicular to the line-of-identity (SD2) before (A) or after the application of 0.1 (B), 0.5 (C), 1 (D) or 2 ​mM heptanol at baseline (E) and 20 ​s before occurrence of ventricular arrhythmias (F).

**Fig. 5:**
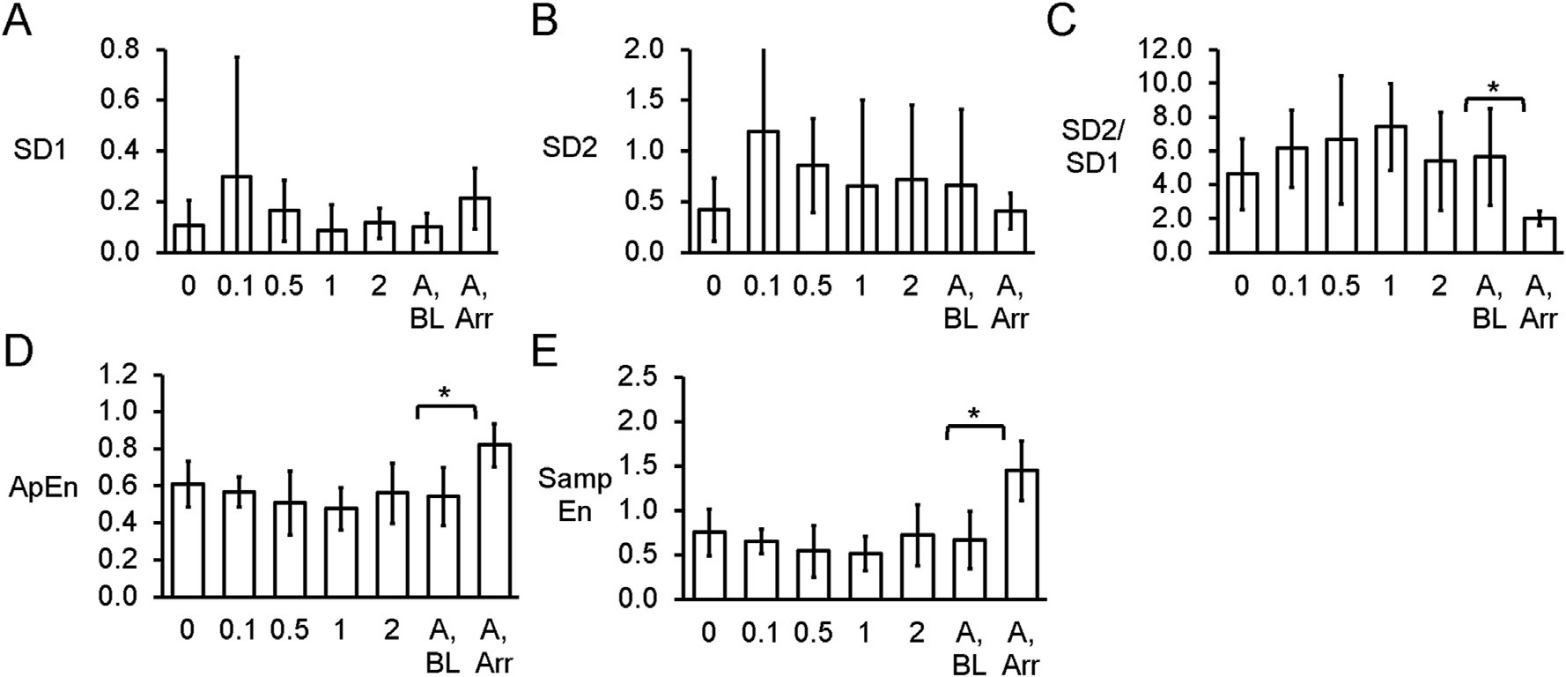
Bar charts plotting SD perpendicular to the line-of-identity (SD1) (A), SD along the line-of-identity (SD2) (B), SD2/SD1 ratio (C), the approximate entropy (D) and the sample entropy (E) (n ​= ​6).

**Fig. 6:**
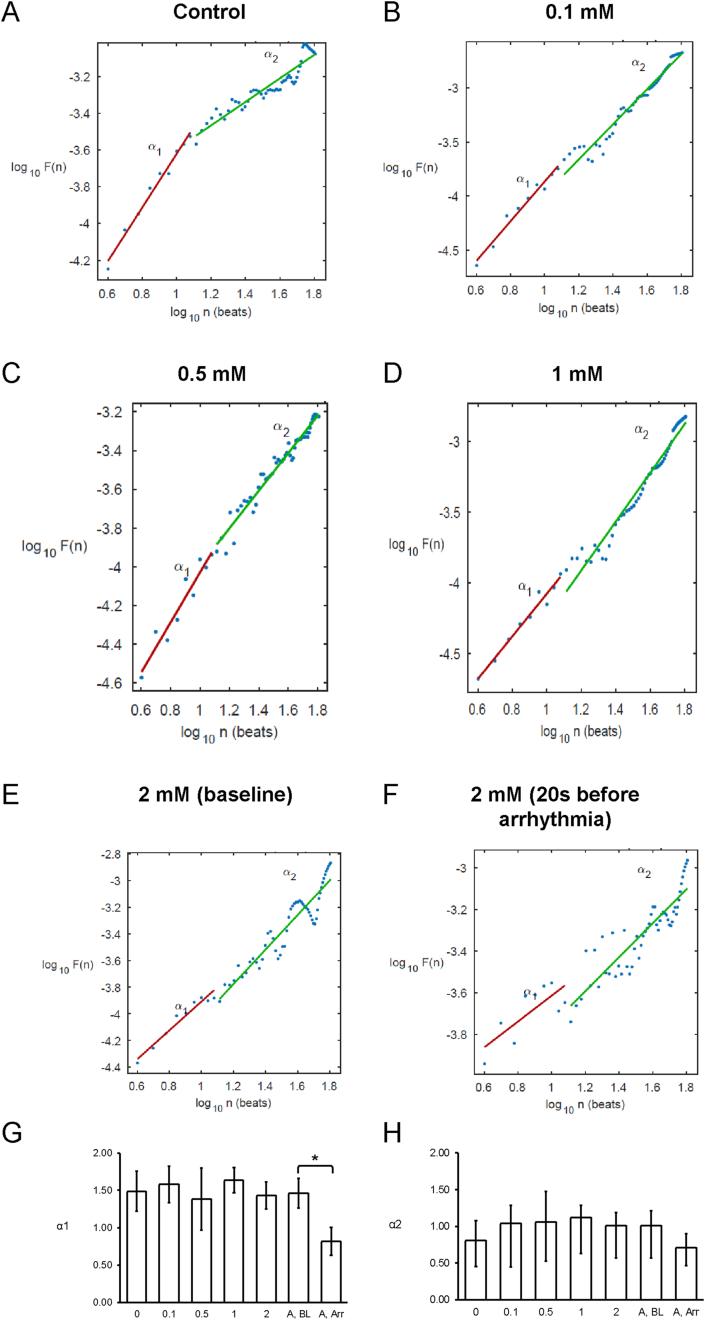
Detrended fluctuation analysis plots expressing detrended fluctuations F(n) as a function of n in a log-log scale before (A) or after the application of 0.1 (B), 0.5 (C), 1 (D) or 2 ​mM heptanol at baseline (E) and 20 ​s before occurrence of ventricular arrhythmias (F). Short-term (G) and long-term (H) fluctuation slopes.

Poincaré plots expressing APD_n+1_ as a function of APD_n_ were constructed (Fig. 4A–E). In all of the hearts studied, ellipsoid shapes of the data points were evident. The SD perpendicular to the line-of-identity (SD1), SD along the line-of-identity (SD2) and the SD2/SD2 ratio are shown in Fig. 5A–C, respectively. The SD2/SD1 ratio was 4.7 ​± ​1.4, whereas approximate and sample entropy took values of 0.38 ​± ​0.06 (Figs. 5D) and 0.53 ​± ​0.15, respectively (Fig. 5E). Detrended fluctuation analysis plotting the detrended fluctuations F(n) as a function of n in a log-log scale was performed (Fig. 6A to E). This revealed short- and long-term fluctuation slopes of 1.33 ​± ​0.50 (Figs. 6F) and 0.65 ​± ​0.37 (Fig. 6G), respectively. None of these parameters described above were altered by heptanol at the concentrations studied during the periods where reproducible MAPs were recorded (KW-ANOVA, *P* ​> ​0.05). By contrast, approximate and sample entropy (0.67 ​± ​0.04 and 0.92 ​± ​0.14; KW-ANOVA, P ​< ​0.05) as well as α1 (0.82 ​± ​0.19; KW-ANOVA, P ​< ​0.05) were significantly increased during the 20 ​s preceding the onset of spontaneous ventricular tachy-arrhythmias.

One heart showed ventricular arrhythmias at 0.5 ​mM. At 2 ​mM heptanol, 5 out of 6 hearts exhibited arrhythmias. For the remaining heart that did not show arrhythmia at 2 ​mM, SD1 and SD2 took values of 0.17 and 0.73, yielding SD2/SD1 of 4.40. The approximate and sample entropy values were 0.65 and 0.97. Short- and long-term fluctuation slopes were 1.43 and 0.69. These values were in between of the values of arrhythmic hearts at baseline and arrhythmic hearts 20 ​s prior to the occurrence of arrhythmias (Supplementary Table 1).

## 4 Discussion

In this study, we examined the effects of varying concentrations of heptanol on beat-to-beat variability in APD and related these to arrhythmic outcomes for the first time in mouse hearts. Our principal findings are that 1) none of the parameters on variability determined using time-domain or non-linear analyses was altered by heptanol during which reproducible MAP activity was observed; 2) in the immediate periods preceding the onset of spontaneous ventricular tachycardia, a decrease in SD2/SD1, increases in approximate and sample entropy and a decrease in short-term fluctuation slope were observed, with the remaining parameters being unaltered.

Previous studies have examined the contributions of heart rate variability using time-domain, frequency-domain and non-linear analyses to arrhythmogenesis (Rajab et al., 2013; Mao et al., 2012; Chen et al., 2008; Cogliati et al., 2002). There are also some studies that have employed similar techniques for investigating beat-to-beat variability in repolarization time-courses, its underlying ionic mechanisms, and relationship to arrhythmogenicity. For example, increased short-term variability in repolarization using the Poincaré plot method was predictive of *torsade de pointes* in dogs (Thomsen et al., 2004). Excessive beat-to-beat variability of repolarization duration is observed during beta-adrenergic stimulation (Johnson Daniel et al., 2013). It was shown that spontaneous Ca^2+^ release contributes to the higher repolarization variability by interspersed APD prolongation, which is exacerbated following potassium channel blockade. Attenuation of Ca^2+^-induced Ca^2+^ release by SCR underlies APD prolongation via increased L-type Ca^2+^ current (Johnson Daniel et al., 2013). A subsequent study by the same group found that beat-to-beat repolarization variability is strongly dependent on sarcoplasmic Ca^2+^ release (Antoons et al., 2015). Furthermore, a combined experimental and computational approach associated higher repolarization variability with pro-arrhythmic abnormalities (Pueyo et al., 2011). These have been translated to assessment of QT variability in humans (Baumert et al., 2016). For example, it can predict pro-arrhythmic outcomes in patients with non-ischemic heart failure (Hinterseer et al., 2010). Recently, we reported in the mouse species for the first time the use of time-, frequency-, time-frequency and non-linear analyses for quantifying APD variability in the atria and ventricles (Tse et al., 2018). Here, we extend these findings by reporting that higher degrees of repolarization variability and irregularity can predict ventricular arrhythmogenesis. However, this study did not examine repolarization variability at the single cell level. It may be that the underlying determinants of repolarization variability is different in mouse compared to other species such as dogs, given the species differences in ion channel types as well as the structure of transverse tubular system (Brette and Orchard, 2003).

Heptanol is known to uncouple gap junctions at low concentrations (<2 ​mM) and also inhibit sodium channels above this concentration. Interestingly, over a range of concentrations studied, we found that it did not alter any of the variability parameters that were determined using time-domain methods. In our previous work, we also reported a lack of effect of this agent on APD time-courses, transmural repolarization gradients, magnitude of APD alternans or restitution slopes (Tse et al., 2016c, 2016d), in contrast to its actions in reducing conduction velocity (CV) and increasing dispersion of conduction. In our previous work, we were unable to pinpoint the mechanism by which ventricular arrhythmias were generated but attributed arrhythmogenesis to conduction abnormalities (Tse et al., 2016c, 2016d). Yet, we were unable to distinguish differences in CVs between arrhythmic and non-arrhythmic groups in the presence of 2 ​mM heptanol. Specifically, heptanol reduced CV to similar degrees despite inducing arrhythmias in some of the hearts studied. This study therefore provides the evidence that variability in repolarization may represent an additional reentrant substrate or at the very least, can be used as a marker of impending arrhythmia. For example, Poincaré plot analysis identified a reduction in SD2/SD1 as a potential biomarker for impending arrhythmogenesis and this warrants further evaluation in future studies. The effects of heptanol on CV reduction are not only dose-dependent (Tse et al., 2016e), but also time-dependent (Tse et al., 2016f), leading to conduction block at higher heptanol concentrations of 1–2 ​mM (Tse et al., 2016c, 2016d). Therefore, a standardized time point of 120 ​s after exposure was selected for the higher heptanol concentrations. Previously, beat-to-beat repolarization variability was assessed over a 60-s period (Tse et al., 2018). However, it was not possible to assess variability over the same timeframe for higher heptanol concentrations, because total conduction block often occurring within a minute leading to distortion and finally abolition of waveforms. Therefore, a shorter duration of 20 ​s was selected.

Previous studies have reported alterations in beat-to-beat repolarization variability with differing levels of gap junction coupling using time-domain methods. Thus, single ventricular cardiomyocytes isolated from canine hearts showed a baseline level of APD variability, which was decreased when two of these cells were electrically coupled (Zaniboni et al., 2000). In modelling studies, a lower level of gap junction coupling was associated with a higher variability (Heijman et al., 2013). In the present work, APD variability assessment using time-analyses was not significantly different after heptanol treatment. There are several possible reasons as to why this may be the case. Heptanol is not a “clean” drug that acts exclusively on gap junctions but can affect other proteins such as potassium and calcium channels. Alterations in the functions of these latter channels may mitigate the effects of gap junction uncoupling alone. Another reason is that the presence of nonstationarities strongly affected results of spectral and complexity analyses (Magagnin et al., 2011). Further studies are needed to explore this issue.

By contrast, the present work clearly associates higher entropy with arrhythmic outcomes, and lower entropy with non-arrhythmic activity. Two methods of calculating entropy were employed. Firstly, approximate entropy was calculated but can bias towards regularity with self-matches (Porta et al., 2007). This problem can be circumvented by means such as calculating sample entropy. Our findings in the mouse are in agreement with clinical studies. For example, in a cohort of forty-seven patients with decreased left ventricular function with implantable cardioverter defibrillators, increases in approximate entropy of the interval between the peak of the R-wave and peak of the T-wave, were independently associated with appropriate ICD shocks and mortality (Perkiomaki et al., 2003). More recently, entropy of QT intervals was reported to predict the occurrence of ventricular arrhythmias and mortality in patients who have received implantable cardioverter-defibrillator for primary prevention of sudden death (DeMazumder et al., 2016). Early detection of ventricular arrhythmias well before the actual event is the biggest challenge in cardiology. Whilst our work reports differences in repolarization variability parameters immediately before the arrhythmias, for the findings to be clinically meaningful, identification of algorithms that can identify subtle changes earlier is required. For example, recent work has reported that ventricular arrhythmias may well be detectable up to 14 ​min before the events occur (Shi et al., 2020). Future studies should explore other methodologies to facilitate early detection.

### 4.1 Limitations

Several limitations should be noted. Firstly, this study was conducted using a small sample size of mice but the techniques underlying this paper have already been validated previously. Some variables such as the long-term fluctuation slope would require larger sample sizes to confirm no real significant difference exists. Secondly, pacing frequency can exert effects on the different variability and regularity measures. Future studies should therefore examine the effects of varying pacing frequency on these parameters. Thirdly, in line with our previous experiments conducted on Langendorff-perfused mouse hearts (Tse et al., 2017), Krebs-Henseleit solution containing 1.8 ​mM calcium chloride was used. However, we recognize that higher calcium concentrations can affect electrophysiology (Tse et al., 2016g) and contractile force (Liao et al., 2012). Whilst other investigators have also used this calcium concentration in their experiments (Georget et al., 2002; Heubach et al., 1999; Basheer et al., 2018), we recognize that this higher calcium concentration can affect Ca^2+^ handling in cardiomyocytes by resetting the Ca^2+^ load of the sarcoplasmic reticulum, which lower threshold for arrhythmogenesis under basal conditions. Fourthly, repolarization variability can be affected by basic cycle length. The relationship between pacing frequency and repolarization variability can be investigated using a dynamic pacing protocol, which delivers regular stimuli over a range of basic cycle lengths. However, we could not apply this protocol for heptanol at higher concentrations, because of the presence of conduction block that occurs before the pacing protocol is completed. However, we have applied this protocol to an acquired long QT mouse model by experimental hypokalaemia (Tse et al., 2021), and the effects of pacing frequency on repolarization variability should be explored in this model. Finally, this study was conducted in a specific pharmacological mouse model. The applicability of these analytical techniques should be extended to different disease and animal models, such as the hypokalaemia model mentioned above, or genetic disease models such as Brugada syndrome in which repolarization variability contributes to the arrhythmic substrate (Tse et al., 2016h; Behar et al., 2017; Bortolan et al., 2009). It should be recognized that there are important differences in the cardiac electrophysiology of mice and humans, for example the different potassium currents responsible for ventricular repolarization (Edwards and Louch, 2017). Larger animals such as horses and dogs may show greater levels of similarities to the human heart (Kaese et al., 2013; Huang et al., 2021). Therefore, future studies with human data are required to extend the generalizability of our findings.

## 5 Conclusions

Measures of repolarization variability, such as SD2/SD1, entropy, and fluctuation slope are altered preceding the occurrence of ventricular arrhythmogenesis in mouse hearts. Changes in these variables may allow detection of impending arrhythmias for early intervention.

## CRediT authorship contribution statement

**Gary Tse:** Conceptualization, Methodology, Software, Formal analysis, Investigation, Resources, Data curation, Writing – original draft, Writing – review & editing, Visualization, Project administration, Funding acquisition. **Guoliang Hao:** Formal analysis, Investigation, Resources, Writing – review & editing. **Sharen Lee:** Formal analysis, Investigation, Writing – review & editing. **Jiandong Zhou:** Methodology, Software, Formal analysis, Investigation, Resources, Data curation, Writing – review & editing. **Qingpeng Zhang:** Resources, Writing – review & editing, Supervision, Project administration. **Yimei Du:** Resources, Writing – review & editing, Supervision, Project administration. **Tong Liu:** Resources, Writing – review & editing, Supervision, Project administration, Funding acquisition. **Shuk Han Cheng:** Resources, Writing – review & editing, Supervision, Project administration, Funding acquisition. **Wing Tak Wong:** Resources, Writing – review & editing, Supervision, Supervision, Project administration, Funding acquisition.

## Declaration of competing interest

The authors declare that they have no known competing financial interests or personal relationships that could have appeared to influence the work reported in this paper.

## Appendix A Supplementary data

Application Application

Application Application
