# Supplementary material for: Measures of repolarization variability predict ventricular arrhythmogenesis in heptanol-treated Langendorff-perfused mouse hearts
Source: Curr Res Physiol. 2021 Apr 19;4:125–34. doi: 10.1016/j.crphys.2021.04.001 (PMC8562203; doi:10.1016/j.crphys.2021.04.001)
Supplement: Application [file mmc1.docx]

**Supplementary Table 1. Comparisons between non-arrhythmic heart at 2 mM, arrhythmic hearts.**

| Heart(s) | SD2/SD1 | Approximate entropy | Sample entropy | Alpha-1 | Alpha-2 |
| --- | --- | --- | --- | --- | --- |
| Non-arrhythmic heart at 2 mM | 4.40 | 0.65 | 0.97 | 1.43 | 0.69 |
| Arrhythmic hearts at baseline | 5.67 ± 2.87 | 0.54 ± 0.16 | 0.67 ± 0.32 | 1.47 ± 0.20 | 1.01 ± 0.44 |
| Arrhythmic hearts 20 seconds before arrhythmias | 2.03 ± 0.41 | 0.82 ± 0.12 | 1.45 ± 0.34 | 0.82 ± 0.19 | 0.71 ± 0.25 |
